# Predictive Factors for Response to Percutaneous Bleomycin in Lymphatic–Venous Malformations of the Head and Neck

**DOI:** 10.3390/jcm14134505

**Published:** 2025-06-25

**Authors:** Thanat Kanthawang, Yuttapol Hirun, Kittisak Unsrisong, Jirapong Vongsfak, Withawat Vuthiwong

**Affiliations:** 1 Department of Radiology, Faculty of Medicine, Chiang Mai University, Chiang Mai 50200, Thailand; thanatkanthawang@gmail.com (T.K.); yuttapol.h@cmu.ac.th (Y.H.); kitlight@hotmail.com (K.U.); jvongsfak@gmail.com (J.V.); 2 Neurosurgery Unit, Department of Surgery, Faculty of Medicine, Chiang Mai University, Chiang Mai 50200, Thailand; 3 Clinical Surgical Research Center, Department of Surgery, Faculty of Medicine, Chiang Mai University, Chiang Mai 50200, Thailand

**Keywords:** lymphatic–venous malformation, bleomycin, predictive factors, head and neck

## Abstract

**Background/Objectives:** This study aims to identify baseline imaging parameters, across various imaging modalities, that can predict the response to bleomycin sclerotherapy in patients with head and neck lymphatic–venous malformations (LVMs). **Methods:** A retrospective analysis of 80 patients (85 lesions) treated at a tertiary care center between January 2018 and December 2022 was conducted. Imaging modalities, including CT, MRI, ultrasonography, and dynamic digital radiographic images, were reviewed for lesion characteristics. Factors including lesion type, volume, morphology, location, and contrast opacification patterns were analyzed for their association with treatment response, defined as a >50% reduction in lesion size and symptom improvement. Univariable and multivariable logistic regression analyses were performed. **Results:** Of 85 lesions, 45 (52.9%) responded to treatment. Univariable analysis showed that pure lymphatic malformations (OR = 6.12, *p* = 0.004), macrocystic components (OR = 10, *p* = 0.016), cavitary morphology on dynamic digital radiographic images (OR = 8.90, *p* < 0.001), neck location (OR = 4, *p* = 0.03), and deep-seated lesions (OR = 3.69, *p* = 0.03) were significantly associated with better outcomes. Multivariable analysis identified cavitary morphology as the strongest predictor (*p* = 0.04). A combination of cavitary morphology, macrocystic components, and pure LM type yielded the highest predictive accuracy (AUC = 0.80, *p* = 0.03). **Conclusions:** The presence of lymphatic channels or large cystic venous spaces—such as macrocystic features on imaging or cavitary morphology—along with neck or deep-seated lesion location, predicts a favorable response to bleomycin sclerotherapy in head and neck LVMs.

## 1. Introduction

Lymphatic–venous malformation (LVM) is a slow-flow vascular malformation, according to the International Society for the Study of Vascular Anomalies [1]. LVM, one of the most common vascular malformations, can manifest in its simple form or in combination [2]. Lymphatic malformation (LM) affects 2.8–5 per 100,000 births; venous malformation (VM) affects 1–5 per 10,000 births [3,4]. Early treatment significantly reduces morbidity, minimizes cosmetic complications, and improves overall patient outcomes [5].

Although sclerotherapy is generally considered the first-line treatment for symptomatic LVMs due to its minimally invasive nature [1,6], there remains no universally accepted guideline that defines specific criteria for treatment selection—including when to choose sclerotherapy over surgery, laser therapy, or combined approaches [7,8]. LVMs often exhibit infiltrative growth, multiple loculations, and proximity to vital structures, making complete excision challenging [5,7]. Consequently, treatment decisions are typically individualized based on lesion characteristics, symptom severity, and institutional expertise. Sclerotherapy technique damages the endothelium, causing inflammation, thrombosis, and fibrosis, resulting in LVM shrinkage [9]. The efficacy of sclerosing agents varies [10,11]. Concentrated ethanol performs more effectively but has more side effects. Bleomycin remains a commonly used option for sclerotherapy due to its favorable safety profile and lower incidence of complications [12]. Our institution’s first-line head and neck LVM sclerosing agent is bleomycin. 

Most LVMs occur in the extremities or head and neck regions and can cause functional impairments, including pain and restricted mobility [2,5]. Head and neck lesions are often associated with more cosmetic concerns and specific risks such as airway obstruction [8]. Sclerotherapy in the head and neck region presents unique challenges due to the difficulty of accessing deep-seated lesions and the dosing limitations of sclerosant agents, particularly in pediatric patients. The results of limited research on predicting factors for LVM percutaneous bleomycin treatment are variable. Additionally, all studies have included LVM cases from various regions with differing anatomical characteristics and technical difficulties [5,9,13,14,15]. 

Imaging plays a crucial role in the characterization and evaluation of LVMs. Magnetic resonance imaging (MRI) and ultrasonography (US) are first-line imaging modalities [1]. Computed tomography (CT) is used to evaluate lesions when MRI is not available. However, specificity in accurately classifying slow-flow vascular malformations is limited. Even with MRI, specificity can be as low as 50%, particularly in cases involving mixed-type vascular malformations [16]. Direct percutaneous contrast administration before sclerotherapy is another peri-interventional imaging modality [17]. Furthermore, LVM imaging parameters predicting responders are controversial [5,9,13,15]. 

Identifying factors that predict response has the potential to improve patient selection and treatment outcomes. This involves decisions about whether to proceed with sclerotherapy, use more sclerosing agents, or undergo early surgery [18,19,20,21]. The recent meta-analyses on LVM in the head and neck treated with bleomycin showed that the overall quality of available studies was low due to substantial heterogeneity and a high risk of bias in allocation, blinding, and incomplete outcome data [22]. This study aims to identify baseline imaging parameters, across various imaging modalities, that can predict the response to bleomycin sclerotherapy in patients with head and neck LVM. 

## 2. Materials and Methods

### 2.1. Study Population

**Figure 1:**
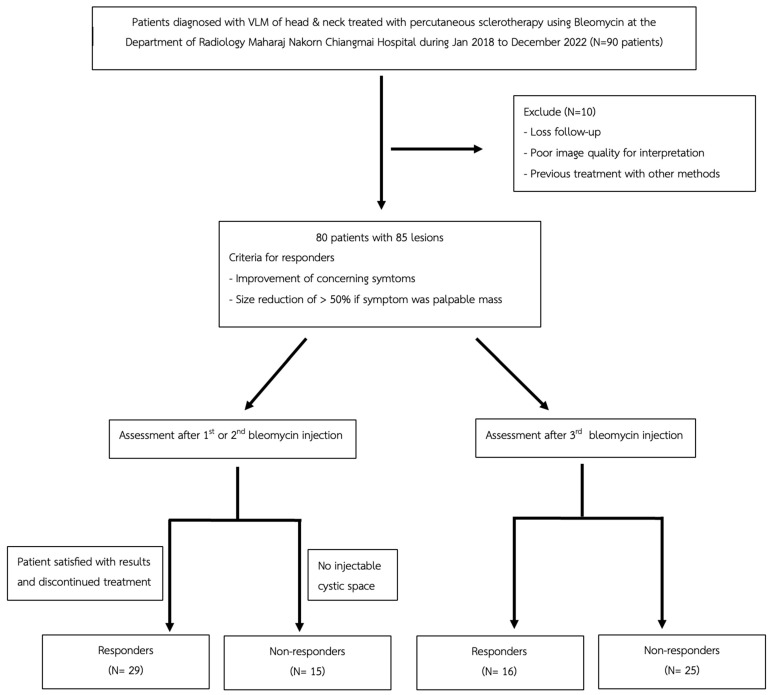
The flow chart of patient selection. LVM = lymphatic–venous malformation.

After Institutional Review Board approval, this retrospective study was conducted in accordance with the Ethics Committee guidelines (Protocol Code: 322/2023, Research ID: 0396, Study Code: RAD-2566-0396). As the data were retrospectively collected, informed consent was waived. All patient data used were anonymized and kept confidential, only accessible to the research team. We retrospectively reviewed 90 consecutive patients with head and neck lymphatic, venous, or mixed-type vascular malformations (LVMs) treated with percutaneous bleomycin sclerotherapy at our tertiary center between January 2018 and December 2022. After exclusions, 80 patients with 85 lesions were included. All had baseline imaging—32 with CT only, 43 with MRI only, and 5 with both—along with peri-interventional ultrasound and dynamic contrast radiography. The patient selection process is summarized in Figure 1.

### 2.2. Final Diagnosis

The diagnosis of LVM was based on clinical symptoms, physical examination, and MRI/CT findings indicating a vascular malformation without arterial shunting. A multidisciplinary team confirmed the diagnosis by correlating these findings with peri-interventional ultrasound, the contrast opacification pattern of dynamic digital radiographic images, and the aspirated content during sclerotherapy—particularly identifying lymphatic fluid for confirming diagnosis of LM or mixed-type malformations [5,9,17,23]. The details of imaging techniques and diagnostic criteria for vascular malformations in each imaging modality are provided in Supplementary Materials.

### 2.3. Treatment Protocol of Bleomycin Sclerotherapy

Informed consent for the sclerotherapy was obtained from all patients following a multidisciplinary team conference that involved various specialists, including an interventional neuroradiologist; a plastic surgeon; an ear, nose, and throat (ENT) specialist; and a nurse. The sclerosing agent used at our institution was exclusively bleomycin, administered by two interventional neuroradiologists who were part of the study.

Procedures were performed under sedation or general anesthesia, determined by the size and location of the lesions. For sedation, midazolam and fentanyl were commonly administered. General anesthesia with endotracheal intubation was utilized for large or deep lesions, particularly those located near critical airway structures. Standard monitoring was employed during all procedures to ensure adequate oxygenation and ventilation.

Selective percutaneous puncture was performed with the guidance of US. The 20–22-gauge needle was used for access to the lesion. After confirming the aspirated content (lymph or blood), the LVMs were injected with iodinated contrast (Optiray 300: Ioversol 300 mgI/mL) under the biplane imaging system (Alphenix Biplane-INFX-8000V, Canon: Canon Medical Systems Coporation, Otawara, Japan) on anteroposterior or oblique view to identify treatable areas. One frame per second is used for the pattern of contrast opacification on dynamic digital radiographic images.

Subsequently, bleomycin (1 mg/mL) was injected at the largest cystic space seen on peri-interventional US and dynamic digital radiographic images. No specific maneuver to occlude the venous outflow during sclerotherapy was performed. The treatment dose was 0.5 mg/kg for pediatric patients, with a maximum dose limit of 15 mg per session for both pediatric and adult patients, and a cumulative dose limit of 100 mg. Therapy completion is indicated by one or more of the following criteria: complete filling of the abnormal channels, lack of aspiration of blood, or the injected sclerosant seen exiting through the needle or cannula left in place [6]. If the dose limit had not been reached, additional injections were administered into other cystic pockets until no further injectable lesions remained. For palpable lesions, treatment was stopped when firm induration indicated complete filling of the lesion. The procedure should be halted if any sign of ischemic change (whitening) is seen in the overlying tissues or there is drainage into normal veins [24].

### 2.4. Treatment Response

Patients underwent physical examinations conducted by an interventional neuroradiologist, with the findings reviewed and confirmed by a multidisciplinary team during regular conferences. These findings were systematically documented in the patients’ medical records at each visit. Follow-up assessments were scheduled at 3-month intervals to monitor treatment response and identify any residual treatable sites. In cases involving deep-seated lesions or inconclusive physical examination findings, follow-up imaging was performed, accounting for 45 lesions (52.94%). The physical examination assessment of LVM improvement included both the actual count and percentage change. For imaging-based follow-up, lesion volume was estimated using the elliptical formula to assess changes over time. Treatment response was categorized according to the criteria set by Yilmaz et al. [25] as worsening, no change, mild to moderate improvement (<50%), or significant improvement (>50%). 

Treatment response was routinely assessed after the third bleomycin injection session or earlier if treatment was discontinued, using standardized outcome measures and reporting protocols as suggested in previous literature [26]. Patients were divided into two groups: responders and non-responders. Responders were defined as patients who showed improvement in their primary symptoms of concern, with these changes confirmed through objective physical examination by two interventional radiologists, along with imaging in cases with inconclusive findings. Non-responders were those whose symptoms persisted or who remained unsatisfied with treatment outcomes, also verified by clinical and/or imaging assessment. If the primary concerning symptom was a palpable mass, a reduction of more than 50% in size after treatment, as determined by physical examination, was classified as a response. 

Of 85 lesions, 44 lesions (51.76%) were treated with single or two bleomycin injections, and 29 lesions defined as responders were those who were satisfied with their treatment results and opted to discontinue sclerotherapy. In the remaining 15 lesions, no injectable cystic space was identified on MRI or US, and those were categorized as non-responders in this analysis (Figure 1).

### 2.5. Data Collection

The clinical data for each patient were retrieved and extracted from electronic medical records. Imaging parameters, including MRI, CT, US, and pattern of contrast opacification on dynamic digital radiographic images, were retrieved and reviewed on a picture archiving and communication system (PACS). Separate lesions in the same patient were independently recorded according to their location. 

The clinical factors include age, gender, location, concerning symptom, and size evaluated by physical examination, as noted in the medical record. The clinical symptoms comprise palpable mass, pain, airway compromise, visual disturbance, dysarthria, bleeding, ptosis, and proptosis. The term “concerning symptom” was defined as the most significant clinical complaint that prompted the patient to seek treatment and served as a key parameter for evaluating clinical response [5,27]. Size by physical examination was also recorded for evaluation of treatment response. The complications after sclerotherapy were also collected from medical records, including fever and flu-like symptoms, post-procedural infection, hyperpigmentation, nausea, vomiting, transient swelling and pain, and pulmonary fibrosis [12]. 

MRI served as the reference imaging modality for patients who underwent both CT and MRI. Of the total cohort, 32 patients underwent CT and 48 underwent MRI. Lesion type (VM, LM, or mixed) was determined based on characteristic CT or MRI findings, supported by peri-interventional ultrasound, the contrast opacification pattern of dynamic digital radiographic images, and the aspirated content during sclerotherapy. The details of diagnostic criteria for vascular malformations in each imaging modality are provided in Supplementary Materials.

The imaging parameters included lesion volume, extension, and other factors. The lesion volume was calculated using the ellipsoidal equation, 0.52 × width × depth × height (in centimeters), for each lesion. Lesion size was measured in T2W-FS for MRI and post-contrast study on CT at baseline. Lesion extension or depth was categorized as superficial, deep, or mixed, with the superficial fascia serving as the reference point. Superficial lesions were located above the fascia, deep lesions extended below it, and mixed lesions contained both superficial and deep components. Other imaging factors included the components (microcystic, macrocystic, or mixed), venous ectasia, phleboliths (low signal intensity focus with susceptibility on gradient echo sequence on MRI [28] or hyperdense calcified thrombus on CT [29], fluid–fluid level, or internal hemorrhage. In detail, the macrocystic component was evaluated only in cases diagnosed as LM or mixed type and consisted of cysts larger than 2 cm, evaluated exclusively by MRI or peri-interventional ultrasound, but not by CT [15,30]. 

**Figure 2:**
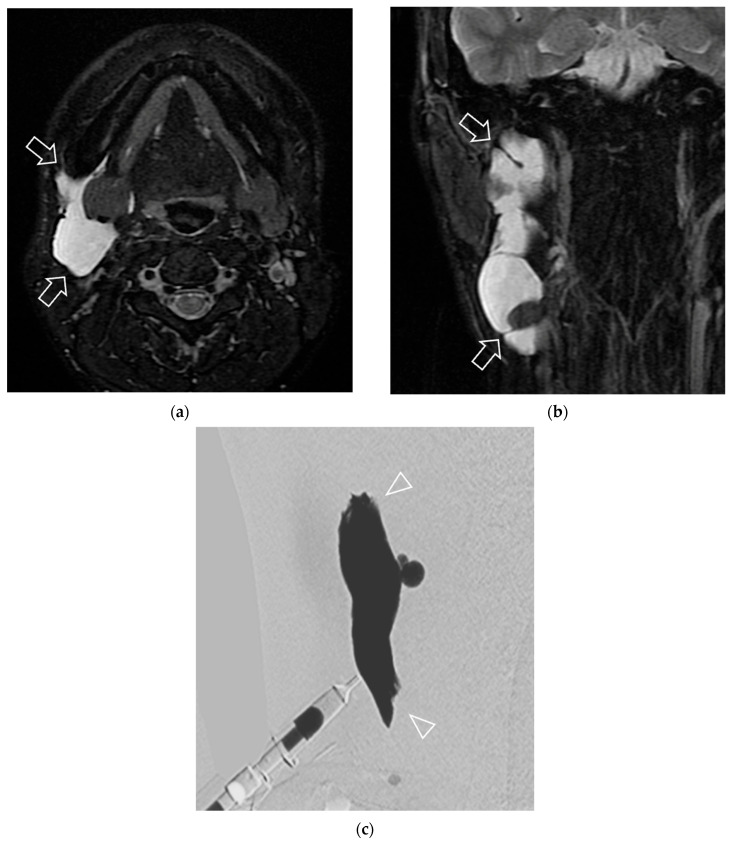
A 21-year-old patient presenting with a palpable mass in the right neck with response to sclerotherapy. (**a**) A pretreatment axial T2-weighted image with fat suppression (**b**) A pretreatment coronal T2-weighted image with fat suppression revealed a lobulated macrocystic lesion with high T2 signal intensity within the right submandibular space or deep-seated lesion (arrows). (**c**) Pattern of contrast opacification on frontal projection of dynamic digital radiographic images corresponding to image b exhibited cavitary morphology (arrowheads). Aspiration of the lesion yielded clear lymphatic fluid. After undergoing one session of bleomycin injection, he experienced improvement in symptoms, with a significant 70% reduction in size confirmed by physical examination and MRI.

**Figure 3:**
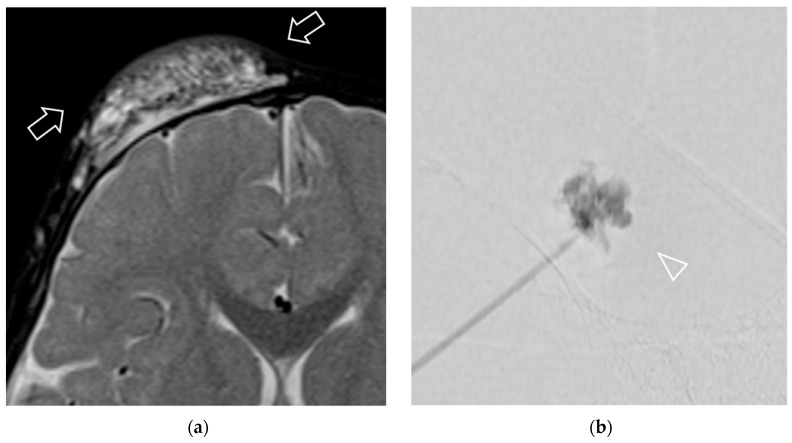
A 12-month-old patient presented with a venous malformation characterized by a palpable mass on the right forehead. The lesion showed non-response to sclerotherapy. (**a**) A pretreatment axial T2-weighted with fat suppression image revealed a microcystic lesion with high T2 signal (arrows) located at the subcutaneous tissue of right frontal scalp. (**b**) Pattern of contrast opacification on dynamic digital radiographic images exhibited spongy morphology (arrowhead), and aspirated contents yielded bloody content. She underwent bleomycin injection but showed no improvement in symptoms and no change in size confirmed by physical examination and MRI after 3 sclerotherapy sessions.

The pattern of contrast opacification on dynamic digital radiographic images was also reviewed for all lesions, including morphology and venous drainage patterns. The morphology was classified into four major categories, according to Bagga et al. 2021 [5], comprising cavitary (discrete, contrast-filled, large, unilocular/multiple cystic spaces), spongy (a cluster of tiny microcystic spaces simulating appearance of sponge), dysplastic vein (a tangle of ectatic abnormal tortuous venous channels), and mixed pattern (any combination of the above mentioned morphologic patterns) (Figure 2 and Figure 3). The venous drainage pattern was adopted from Puig et al. 2003 [31], divided into Type I (isolated malformation without venous drainage), Type II (malformation with drainage into normal veins), Type III (malformation with drainage into dilated veins), and Type IV (dysplastic venous ectasia) (Figure 2 and Figure 3). The presence of phleboliths was also recorded during dynamic digital radiographic images, along with CT or MRI findings. 

All imaging parameters were independently reviewed by one interventional radiologist with 8 years of experience and a third-year radiology resident, both of whom were blinded to clinical information. In cases of disagreement, a final decision was made under the supervision of an interventional radiologist with 16 years of experience and a musculoskeletal radiologist with 10 years of experience. A training session was conducted for both reviewers using cases not included in this study to minimize bias. 

### 2.6. Statistical Analysis

Statistical analysis was performed using STATA statistical software, version 17. The critical level of statistical significance was set at a *p*-value of less than 0.05. Data were summarized descriptively using the median and interquartile range (IQR) for continuous data, as the data were not normally distributed, and frequencies and percentages for categorical data. Univariable and multivariable logistic regression were used to identify potential imaging predictors for responders after bleomycin sclerotherapy. 

Subgroup analysis was performed for significant univariable factors related to the total number of treatments, categorized into three groups: 1, 2, and ≥3 sclerotherapy sessions. Subgroup analysis was also performed for significant univariable factors in lesions presenting with a palpable mass. Additionally, a subgroup analysis was conducted for significant univariable factors based on the type of LVM, with venous malformations (VM), lymphatic malformations (LM), and mixed-type lesions analyzed separately. Inter-reader reproducibility of imaging parameters was assessed using intraclass correlation coefficients (ICC) [32]. 

## 3. Results

Among 80 patients with 85 separate lesions, the median age was 14 years (range 1–69 years), with 56.47% being female. Five out of 80 patients (6.25%) had two concerning lesions, with the treatment response assessed for each lesion. Table 1 shows patient characteristics in this study. The median volume of LVM was 31.6 mL (IQR: 14.7–65.1 mL). Of the 85 lesions, 45 (52.94%) were classified as responders. Among the 85 lesions, A palpable mass was the most common presenting symptom, observed in 66 lesions (77.65%), followed by pain in 9 lesions (10.58%). There was no statistically significant difference in symptom distribution between the responder and non-responder groups.

In the responder group (45 lesions), all cases with a concerning palpable mass (43 lesions, 95.56%) showed a >50% reduction in size after treatment. The remaining three lesions in the responder group were categorized based on improvements in dysarthria, discoloration, airway compromise, and proptosis. Non-responders (40 lesions) showed no significant volume reduction, with 6 lesions (15%) worsening, 18 lesions (45%) showing no change, and 14 lesions (35%) having a <50% reduction in size. The remaining 2 of the 40 lesions were categorized based on the absence of symptom improvement in the tongue and oropharynx due to mass effect. 

LVM location was significantly associated with treatment response, with a higher proportion of neck lesions in the responder group compared to non-responders (44.44% vs. 12.50%, *p* = 0.002). The responder group also showed a slight female predominance (64.44% vs. 47.50%), though there were no statistically significant differences between the two groups in terms of age or sex.

Univariable analysis revealed statistical significance for the location of the lesion, type of LVM, component, and pattern of contrast opacification on dynamic digital radiographic images (Table 2, Figure 2 and Figure 3). Specifically, pure LM showed a significantly greater response compared to pure VM (odds ratio (OR) = 6.12, 95% CI: 1.88–19.92, *p* = 0.004). A mixed type of VM and LM showed an OR of 2.93 (95% CI: 0.99–8.64) compared to pure VM, but this finding did not reach statistical significance (*p* = 0.052). Regarding the component in LM and mixed LVM (43 lesions), the macrocystic component showed a significantly greater response than the microcystic component (OR = 10, 95% CI: 1.54–64.75, *p* = 0.016). For the pattern of contrast opacification on dynamic digital radiographic images, only the cavitary morphology showed a significantly greater response than the spongy morphology (OR = 8.90, 95% CI: 3.11–25.45, *p* < 0.001). Neck location (OR = 4.00, 95% CI: 1.17–13.66, *p* = 0.03) and deep-seated lesions (OR = 3.69, 95% CI: 1.00–10.00, *p* = 0.03) were significantly associated with the responder group.

Multivariable analysis identified cavitary morphology in the pattern of contrast opacification as the only significant predictor (*p* = 0.04). Subgroup analysis of statistically significant imaging parameters—including lesion location in the neck, lesion depth, and cavitary morphology—was performed separately for cases with LM and VM. The results showed that only cavitary morphology on dynamic digital radiographic imaging remained significant, with an odds ratio of 5.6 (95% CI: 1.67–18.74; *p* = 0.005) for VM and 9.2 (95% CI: 1.97–42.97; *p* = 0.005) for LM. We also performed a subgroup analysis focusing solely on patients whose primary concern was a palpable mass, and the results remained consistent.

**Figure 4:**
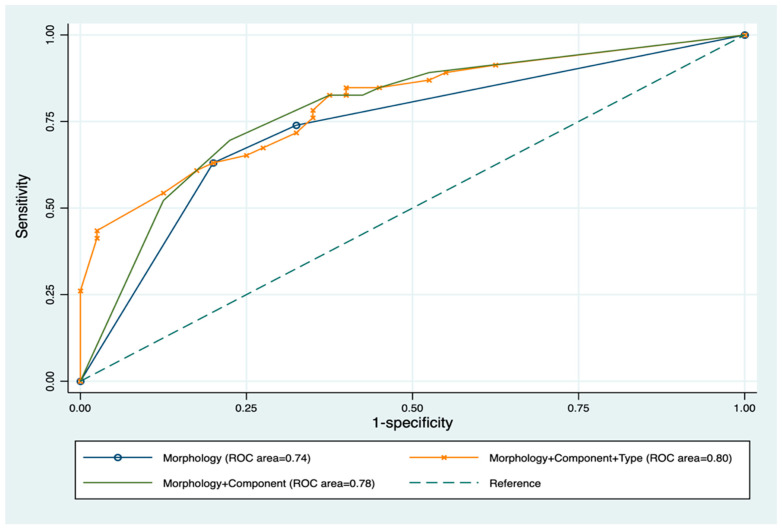
Correlation between significant imaging and pattern of contrast opacification on dynamic digital radiographic images of LVM and responder from sclerotherapy. The AUC for morphology alone is 0.74 (95% CI, 0.64–0.83). The AUC for combination of cavitary morphology and macrocystic component is 0.78 (95% CI, 0.69–0.88). The AUC for combination of cavitary morphology, macrocystic component, and LM is 0.80 (95% CI, 0.71–0.89). AUC = area under the ROC (receiver operating characteristic) curve.

The accuracy of prediction using a combination of significant imaging parameters was compared to using morphology alone [AUC = 0.74 (95% CI, 0.64–0.83)] (Figure 4). The AUC increased to 0.78 for the combination of cavitary morphology and macrocystic component (*p*-value < 0.001) and further increased to 0.80 for the combination of cavitary morphology, macrocystic component, and pure LM type (*p*-value = 0.03). The remaining combinations showed no statistical significance. 

The median number of sclerotherapy sessions performed on these patients was three (IQR: 3–5.5) for non-responders and two (IQR: 1–4) for responders (*p*-value = 0.01). The median dose of bleomycin per session was 12 mg for responders and 10 mg for non-responders. The follow-up period ranged from 3 months to 9 years, with a median duration of 1.5 years. A complication occurred in one patient (1.23%), who developed redness and swelling after the second treatment session, which resolved spontaneously. No patients in our cohort experienced permanent skin changes or pulmonary complications related to the treatment.

In the subgroup analysis of significant univariable factors related to the total number of treatments, divided into three groups (1, 2, and ≥3 sclerotherapy sessions), pure LM tended to be treated less often than pure VM (8/3/6 for pure LM and 1/7/8 for pure VM in the 1/2/≥3 times of sclerotherapy, respectively). However, this difference did not reach statistical significance (*p*-value = 0.063). The remaining factors also showed no statistical significance. 

The interobserver reliability for each imaging factor was calculated and showed almost perfect agreement for component, phlebolith, morphology, and venous drainage (ranging from 0.91 to 1.00 for ICC), but moderate agreement for extension, venous ectasia, and fluid–fluid level (ranging from 0.64 to 0.75 for ICC).

## 4. Discussion

In the head and neck LVM cases of this study, neck location, pure LM, deep-seated lesion, macrocystic components in LM or mixed LVM, and cavitary morphology at baseline imaging were associated with responders to percutaneous bleomycin, particularly when occurring in combination. Conversely, pure VM, superficial lesion, microcystic components, and spongy morphology were predictive of a poor response. Cavitary or spongy morphology in the pattern of contrast opacification on dynamic digital radiographic images alone could predict the response. The interobserver reliability of these imaging features ranged from moderate to almost perfect. 

Two recent meta-analyses of bleomycin sclerotherapy for LVM in the head and neck reported a reduction in patient symptoms ranging from 84% to 96.3% for LM and LVM, respectively, with success rates varying widely from 39% to 100% [11,30]. The effectiveness of percutaneous bleomycin sclerotherapy varies across studies due to differences in treatment response criteria, imaging modalities, vascular malformation types, locations, and sclerotherapy techniques, highlighting the need for more validated studies. 

Our study showed a slightly lower response rate (53.5%), but it remained within the reported range. These results can partly be explained by variations in treatment response criteria noted in the meta-analyses, emphasizing the need for standardized studies with validated outcome measures. While size reduction is commonly measured in studies, its correlation with symptom relief and patient satisfaction remains uncertain [12]. Patient-reported outcome measures, as used in our study, might provide more accurate evaluations of treatment response [12,30,33]. 

Imaging features have been identified as effective predictors of response to bleomycin sclerotherapy. However, these findings are primarily based on LVM in the body, which has different treatment decision-making processes compared to lesions in the head and neck, leading to conflicting results [5,9,13,14,15]. In the head and neck, cosmetic and functional concerns often necessitate more cautious decision making, while body lesions typically focus on function and mobility. Techniques like venous flow occlusion are also more feasible in the extremities. Moreover, the most accurate imaging modalities for predicting treatment response remain unclear. Early detection of LVM lesions, whether responsive or unresponsive to sclerotherapy, can aid in appropriate patient selection. Patients showing a positive response may benefit from additional sclerotherapy sessions, while poor responders may require alternative sclerosants, cryotherapy, or early surgical intervention [18,19,20,21]. There is variation in clinical practice, with some centers using bleomycin alone, while others combine it with different sclerosing agents. Identifying predictive imaging factors can help guide the selection of the most suitable sclerosing agent. The imaging parameters assessed in this study were based on findings from previous research [5,9,13,15]. 

The pattern of contrast opacification on dynamic digital radiographic images not only supports the diagnosis of LVM—especially in combined-type cases and without available pre-treatment MRI—but also aids in identifying potential treatment spaces for sclerotherapy, as demonstrated in our multivariable analysis [2]. While Bagga et al. included morphology in their analysis of dynamic digital radiographic images, they did not find a significant correlation with bleomycin sclerotherapy response [5]. They also reported that the presence of a draining vein and dysplastic vein morphology correlated with a poor response [5], but this association was not observed in our study or others [9,13,14]. These conflicting results may be attributed to differences in study populations, as prior studies included LVM in different anatomical locations. In our experience, smaller venous drainage and limited post-sclerotherapy compression techniques in head and neck LVM may contribute to these findings. Our results support contrast opacification as the most effective imaging modality for evaluating LVM and guiding sclerotherapy response, as it can be applied across all LVM types [34]. This highlights the importance of recognizing the distinct characteristics of LVM in different locations and underscores the need for routine percutaneous contrast opacification before sclerotherapy. 

The response to bleomycin sclerotherapy varies among different vascular malformation types. Our study found that LM and mixed types had more favorable responses than VM, in line with previous studies [5,27]. Meta-analyses focusing on bleomycin sclerotherapy reported significant size reduction rates of 84% for LM and 87% for VM [12]. However, Churojana A et al. [9], who used either ethanol or bleomycin, reported a higher response rate for VM compared to LM (49.2% and 21.4%, respectively). The inclusion of LVM from various locations may contribute to these discrepancies. The higher prevalence of lymphatic malformations (LMs), particularly the macrocystic type, in the head and neck region compared to the extremities may help explain these findings [35,36]. Moreover, in the head and neck, cosmetic and functional concerns often necessitate more cautious decision making, while body lesions typically focus on function and mobility. Techniques like venous flow occlusion are also more feasible in the extremities. Subgroup analysis in our study suggests that pure VM may require more treatment sessions for a good response compared to pure LM, with the latter showing greater responsiveness after 1–2 sessions. Our results support the idea that LM has a greater response than VM. 

Pure LMs most commonly occur in the head and neck region, accounting for approximately 70–80% of cases, particularly within the posterior cervical triangle of the neck [37]. The higher prevalence of LMs in the neck is likely attributable to the rich lymphatic network in this area [38]. In contrast, pure VMs are more broadly distributed, with 60% involving the trunk and extremities and the remaining 40% affecting the head and neck region without a specific predilection for any anatomical space [37]. Moreover, deep-seated LMs most frequently affect the lower two-thirds of the head and neck region [38]. These findings support our results, suggesting that anatomical location—particularly the neck and deep facial regions—is associated with a favorable response to bleomycin sclerotherapy, likely reflecting the higher prevalence of LMs in these areas.

The macrocystic component in LM or mixed-type LVM has been associated with better response to bleomycin sclerotherapy, whereas the microcystic component is linked to non-responders [5,15,18]. The smaller cystic spaces in microcystic lesions hinder sclerosant diffusion, resulting in less lesion size reduction compared to macrocystic lesions. MRI is the preferred imaging modality for assessing macrocystic lesions, although ultrasound (US) can also provide accurate evaluations [39]. In this study, for patients without MRI, we used peri-interventional US to assess macrocystic lesions. We also observed that macrocystic components frequently appear as cavitary patterns on dynamic digital subtraction images. For large-volume lymphatic malformations (LMs), particularly those exceeding 15 mL, sclerotherapy is the preferred first-line treatment due to its minimally invasive nature and favorable outcomes. Bleomycin is commonly used for its safety and efficacy, especially when multiple sessions are required, although strict dose control is essential in large cystic lesions to minimize systemic absorption [40]. Our findings support this approach, showing no statistically significant difference in median pre-treatment volume between non-responders and responders (35.81 mL vs. 31.57 mL, respectively). Surgical resection may be considered after significant volume reduction, particularly in cases involving airway or digestive tract compromise in the infrahyoid or suprahyoid regions [7], but remains challenging in the head and neck due to anatomical complexity and recurrence rates of up to 40% [41]. Alternative sclerosants such as polidocanol, doxycycline, and ethanol have shown varying efficacy but are limited by complications including local inflammation, neurotoxicity, and systemic toxicity [42,43,44,45]. Sirolimus has demonstrated promise for complex or refractory LMs, especially in pediatric head and neck cases, but its use should be guided by a multidisciplinary team. Ultimately, treatment should be individualized based on lesion characteristics, with bleomycin continuing to serve as a safe and effective mainstay [46].

**Figure 5:**
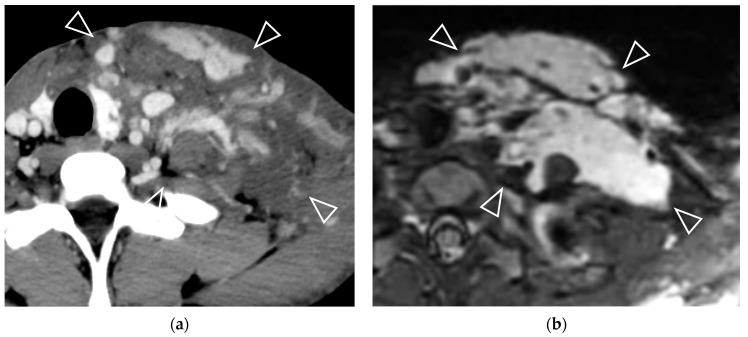
A 10-year-old boy with an infiltrative venous malformation on the left side of his neck. (**a**) A post-contrast CT neck showed an infiltrative hypodense lesion with incomplete contrast enhancement (arrowheads). (**b**) Axial T2-weighted with fat suppression image showed a multiloculated cystic lesion with high signal intensity involving multiple compartments of the left-sided neck (arrowheads). Following the third session of bleomycin injection, the patient observed an improvement in symptoms, accompanied by a reduction in size of over 50%.

Previous studies have included LVM in the extremities and the body, along with the head and neck, and have shown that diffuse involvement is a poor predictor of response to bleomycin sclerotherapy [5,9,13,15,47]. Diffuse involvement, defined as ill-defined borders or extension into more than one body region or compartment. However, we did not include this imaging feature in our study, as it varies by lesion type; LMs generally have more well-defined margins than VMs or mixed types. Moreover, based on our experience, we observed that the margins of most vascular malformations in the head and neck show well-defined borders on MRI, even when they involve multiple compartments (Figure 5). Conversely, the margins of vascular malformations on CT scans are mostly ill-defined (Figure 5). This variability limits the utility of this feature in routine clinical practice, particularly when MRI is not available for review.

This study has several limitations. First, its retrospective design and relatively small sample size limited the statistical power for subgroup analyses based on specific lesion types or primary symptoms. However, our aim was to generalize the findings to all LVMs, reflecting real-world clinical practice. Second, incomplete imaging data could have affected our findings. Only 60% of patients underwent pre-treatment MRI—the imaging modality of choice for LVM diagnosis. Nevertheless, all patients received pre-procedural US and dynamic digital radiographic imaging with contrast opacification, and diagnoses were further supported by the nature of aspirated content during the procedure. Advanced MRI techniques, including dynamic contrast-enhanced studies, were not available. This technique could have improved lesion characterization, volumetric accuracy, and perfusion assessment [16,48]. Additionally, only half of the patients with palpable lesions underwent follow-up imaging. Lesion volume was estimated using the ellipsoidal formula, which may be suboptimal for irregular or infiltrative lesions. However, these limitations reflect routine clinical practice, where MRI access may be limited and standardized imaging protocols for LVM are lacking. To mitigate some of these limitations, response was defined as a >50% reduction in lesion size based on physical examination—primarily for superficial lesions. Future studies incorporating standardized MRI follow-up and volumetric analysis using dedicated [49]. Third, compressibility was not assessed on ultrasound, which could have helped identify thrombotic venous malformations associated with poor treatment response. Fourth, there was some heterogeneity in outcome definitions, as treatment response was based on improvement in the patient’s primary concern—ranging from size reduction to pain relief. While we excluded inappropriate outcomes like skin discoloration and performed a subgroup analysis using volume reduction, this variability may still introduce bias. Lastly, we were unable to evaluate certain clinical predictors such as elevated aPTT or thrombocrit [50]. Future larger, prospective studies with standardized imaging protocols and outcome measures are needed to validate our findings.

## 5. Conclusions

The presence of lymphatic channels or large cystic venous spaces is a strong predictor of treatment response. Features such as macrocystic spaces on MRI or ultrasound, cavitary morphology on dynamic digital radiographic images, as well as lesions located in the neck or deep-seated regions, are all associated with a favorable response to bleomycin sclerotherapy in head and neck LVM.

## Figures and Tables

**Table 1:** Patient characteristics.

Characteristics	Number of Patients (*n* = 80 Patients/85 Lesions)		*p*-Value **
	Non-Responders (*n* = 40)	Responders * (*n* = 45)	
Age at first treatment (years)	14.5 (6.5–38)	14 (3–36)	0.13
Female	19 (47.50%)	29 (64.44%)	0.11
Location			0.002
Neck	5 (12.50%)	20 (44.44%)	
Cheek	14 (35%)	14 (31.11%)	
Scalp	4 (10%)	3 (6.67%)	
Lip	6 (15%)	2 (4.44%)	
Eyelid and orbit	4 (10%)	1 (2.22%)	
Others ***	5 (12.50%)	5 (11.12%)	
Concerning symptom			0.001
Mass	32 (80%)	43 (95.56%)	
Pain	4 (10%)	0	
Others ****	4 (10%)	3 (6.67%)	
Change in size by physical examination with or without imaging			<0.001
Worsening	6 (15%)	0	
No change	18 (45%)	1 (2.22%)	
Size reduction < 50%	14 (35%)	2 (4.44%)	
Size reduction > 50% *****	0	43 (95.56%)	

Values are given as *n* (%) or median (IQR). * Responders were defined by resolution of concerning symptom. ** *p*-value: Fisher exact test or Wilcoxon rank-sum (Mann–Whitney) test. *** Other locations include tongue, nasal ala, and oropharynx. **** Other symptoms include airway compromise, visual disturbance, dysarthria, bleeding, ptosis, and proptosis. ***** Lesions with size reduction > 50% were classified as significant size reduction according to Yilmaz et al. [21].

**Table 2:** Univariable and multivariable analysis of imaging parameters.

Variables	Response		Univariable Analysis		Multivariable Analysis *	
	Non-Responders (*n* = 40)	Responders (*n* = 45)	OR (95% CI)	*p*-Value	OR (95% CI)	*p*-Value
Location at neck	5 (12.50%)	20 (44.44%)	4 (1.17–13.66)	0.03	3.09 (0.53–17.98)	0.21
Type of LVM						
Pure venous malformation	27 (67.50%)	15 (33.33%)				
Pure lymphatic malformation	5 (12.50%)	17 (37.78%)	6.12 (1.88–19.92)	0.004	3.43 (0.40–14.85)	0.34
Mixed	8 (20.00%)	13 (28.89%)	2.93 (0.99–8.64)	0.052	1.54 (0.30–7.84)	0.60
Imaging features						
Pre-treatment volume (mL) **	31.57 (14.79–55.27)	35.81 (15.04–66.20)	1.00 (0.99–1.00)	0.64	1.00 (0.99–1.01)	0.81
Depth of lesion ***						
Superficial	12 (30.00%)	6 (13.33%)				
Deep	13 (32.50%)	24 (53.33%)	3.69 (1.00–10.0)	0.03	3.74 (0.66–21.25)	0.14
Mixed	15 (37.50%)	15 (33.33%)	2.00 (0.59–6.73)	0.26	0.59 (0.09–3.95)	0.59
Component in LM	(*n* = 13)	(*n* = 30)				
Microcystic	5 (38.46%)	2 (6.67%)				
Macrocystic	8 (61.54%)	28 (93.33%)	10 (1.54–64.75)	0.016	3.74 (0.66–21.25)	0.14
Presence of venous ectasia	7 (17.50%)	5 (11.11%)	0.59 (0.17–2.03)	0.40	0.31 (0.02–5.22)	0.42
Presence of fluid level/ hemorrhage	0	3 (6.67%)	1.05 (0.68–1.62)	0.83	*	*
Presence of phlebolith	15 (37.50%)	9 (20.00%)	2.46 (0.93–6.51)	0.077	0.26 (0.04–1.60)	0.15
Pattern of contrast opacification on dynamic digital radiographic images						
Morphology						
Spongy	27 (67.50%)	11 (24.44%)				
Cavitary	8 (20.00%)	29 (64.44%)	8.90 (3.11–25.45)	<0.001	5.90 (1.05–33.05)	0.04
Mixed	5 (12.50%)	5 (11.11%)	3.63 (0.84–15.70)	0.09	5.18 (0.57–47.55)	0.15
Type of venous drainage ****						
Type I	25 (62.50%)	32 (71.11%)				
Type II	10 (25.00%)	11 (24.44%)	0.86 (0.35–2.34)	0.77	4.30 (0.94–19.75)	0.06
Type III	5 (12.50%)	2 (4.44%)	0.31 (0.06–1.75)	0.19	3.08 (0.15–63.46)	0.47

Values are given as *n* (%) or median (IQR). * Multivariable analysis cannot be evaluated. ** Pre-treatment size was calculated by using ellipsoid equation (width × depth × height × 0.52). *** Superficial and deep locations were differentiated by using superficial neck fascia as reference. **** Type I = isolated malformation without venous drainage, Type II = malformation with drainage into normal veins, Type III = malformation with drainage into dilated veins, Type IV = dysplastic venous ectasia. Abbreviations: OR = odds ratio, ROC = receiver operating characteristic curve, LVM = lymphatic–venous malformation, mL = milliliter.

## Data Availability

The data presented in this study are available upon request from the corresponding author. The data are not publicly available due to identity reasons.

## Supplementary Materials

The following supporting information can be downloaded at: https://www.mdpi.com/article/10.3390/jcm14134505/s1.
