# Supplementary material for: Predictive Factors for Response to Percutaneous Bleomycin in Lymphatic–Venous Malformations of the Head and Neck
Source: J Clin Med. 2025 Jun 25;14(13):4505. doi: 10.3390/jcm14134505 (PMC12249929; doi:10.3390/jcm14134505)
Supplement: Supplementary file 1 [file jcm-14-04505-s001.zip › jcm-3597430-supplementary.pdf]

Supplementary Materials

Imaging techniques

MRI and MR angiography (MRA)

All patients underwent MRI on a 3 Tesla SIGNA Pioneer (GE HealthCare, Waukesha, Wisconsin, USA) with 21-channel head and neck coils. The MRI protocol included the axial plane with a 4 mm slice thickness for T1-weighted (T1W), fat-suppressed T2-weighted (T2W-FS), and contrast-enhanced fat-suppressed T1W images with a 5–10-minute delayed phase. The coronal plane was acquired with a 3 mm slice thickness for T1W, T2W-FS, and contrast-enhanced fat-suppressed T1W images. All MRI studies used both MRA methods, including Time-of-Flight (TOF) MRA and contrast-enhanced MRA.

Pre and post contrast CT

The study utilized multidetector CT scanners SOMATOM Force (Siemens Healthineers, Forchheim, Germany) to perform pre- and post-contrast CT scans. The scans consisted of 384 slices (2x192) and used 5-mm and 1.5-mm thick axial sections for pre-contrast CT, post-contrast CT, and a 10-minute delayed scan, along with 3-mm thick coronal or sagittal reconstructions of the contrast-enhanced CT. No patient underwent CT angiography.

Imaging interpretation for each type of LVM

On MRI, VMs present as well-defined, lobulated lesions with high T2 and low T1 signal intensities, showing gradual contrast enhancement. On CT, VMs appear as low-density lesions with slow enhancement, often accompanied by phleboliths. LMs, on the other hand, appear on MRI as multilocular cystic structures with high T2 and low T1 signal with only septal enhancement on post-contrast study. On CT, LMs are seen as low-attenuation cystic spaces with only septal contrast enhancement. A mixed type of VM and LM was classified if the lesion contained both patterns of contrast enhancement on either CT or MRI.
